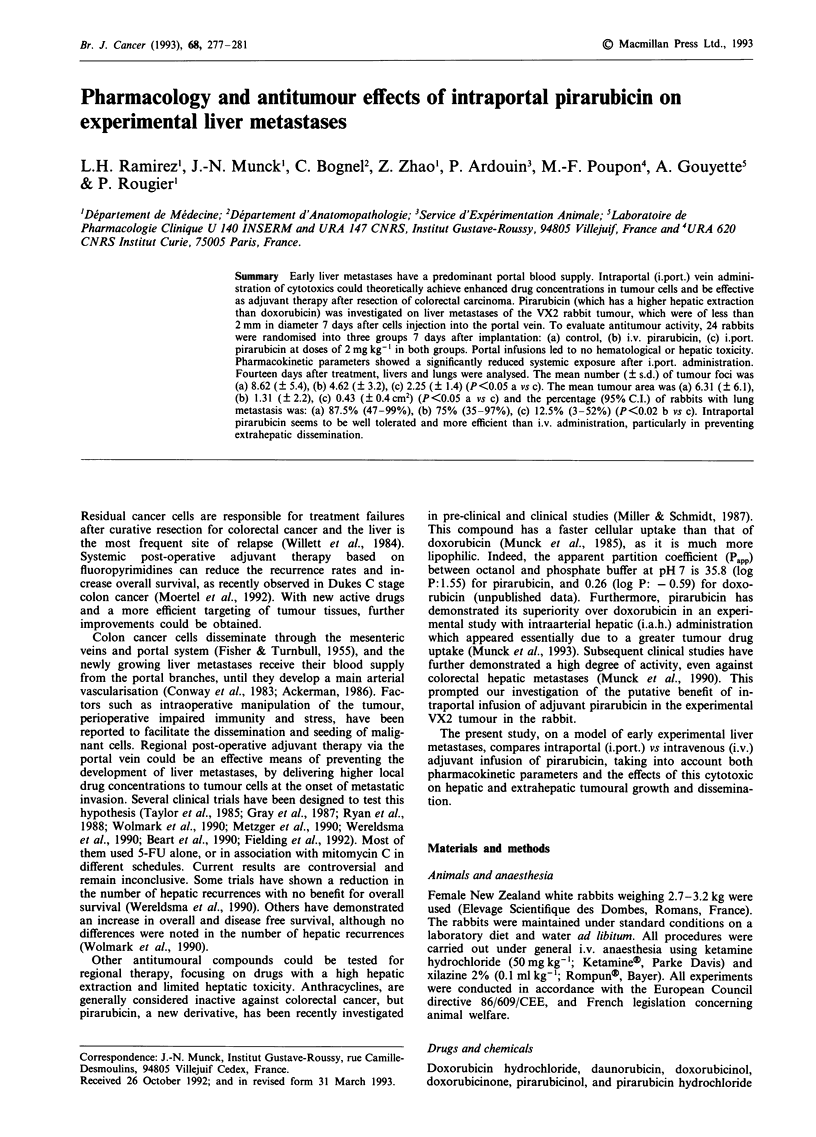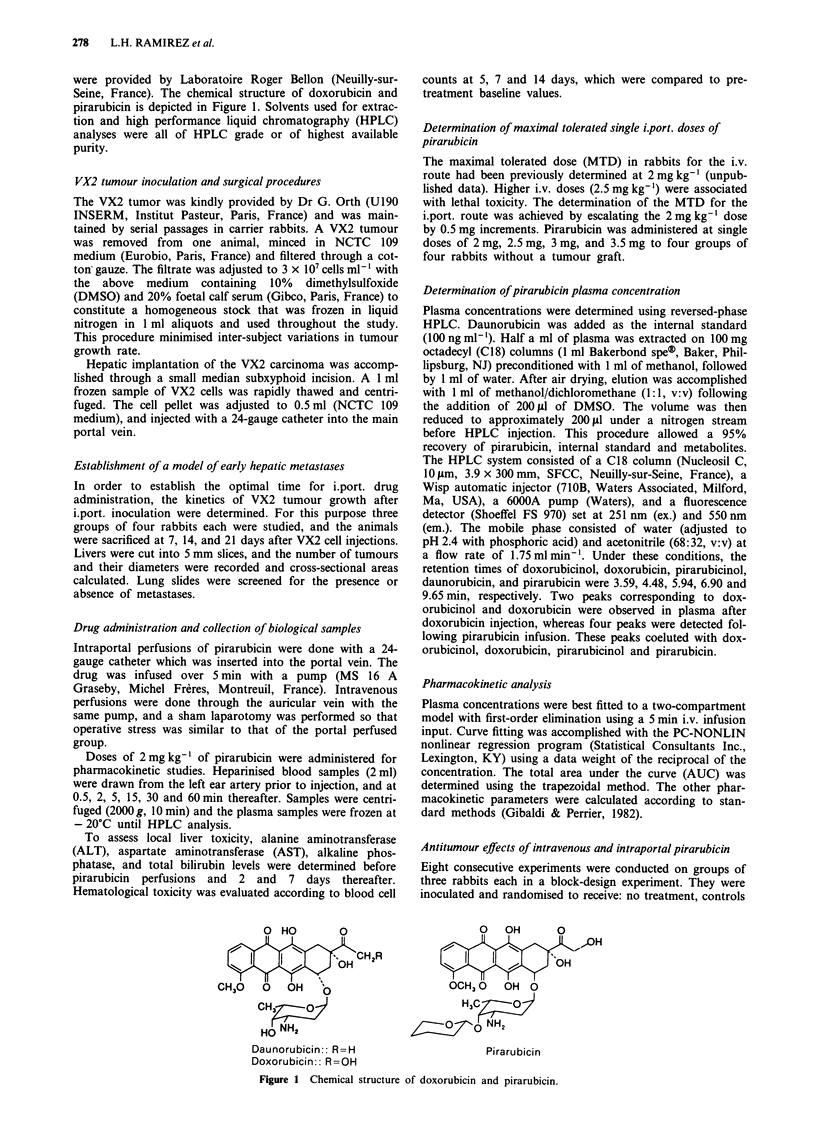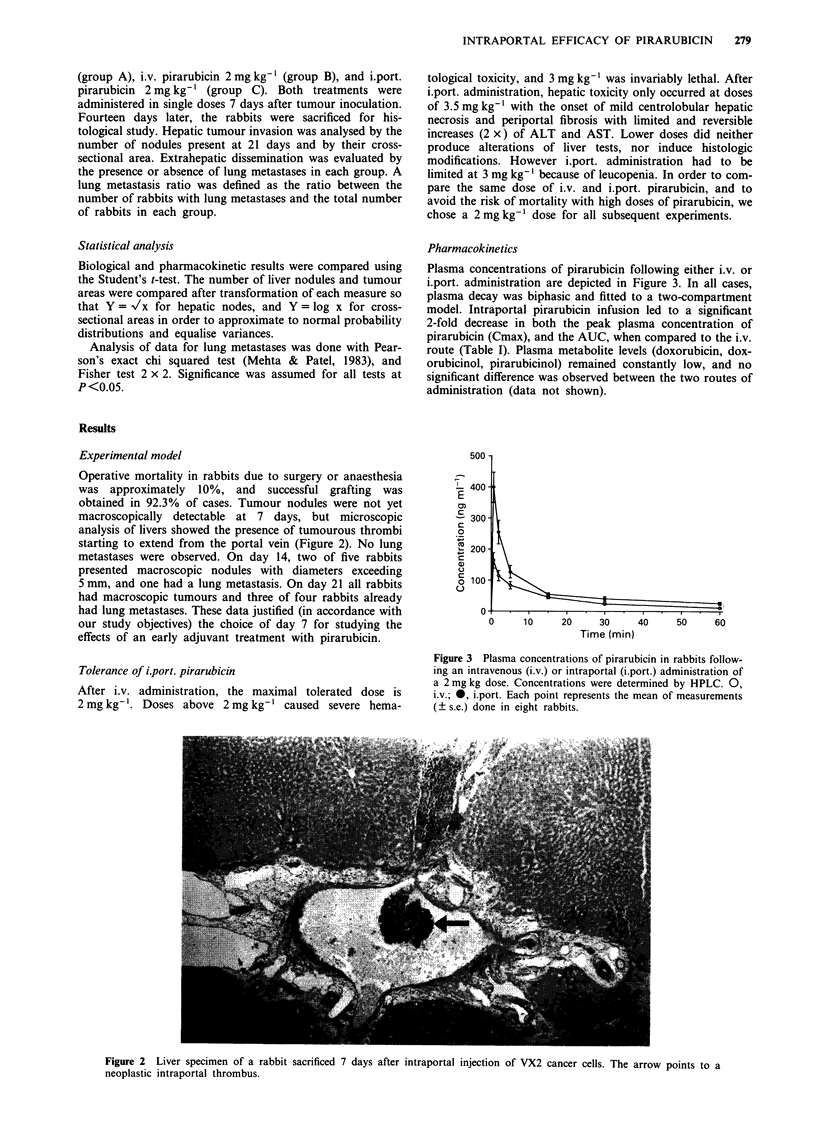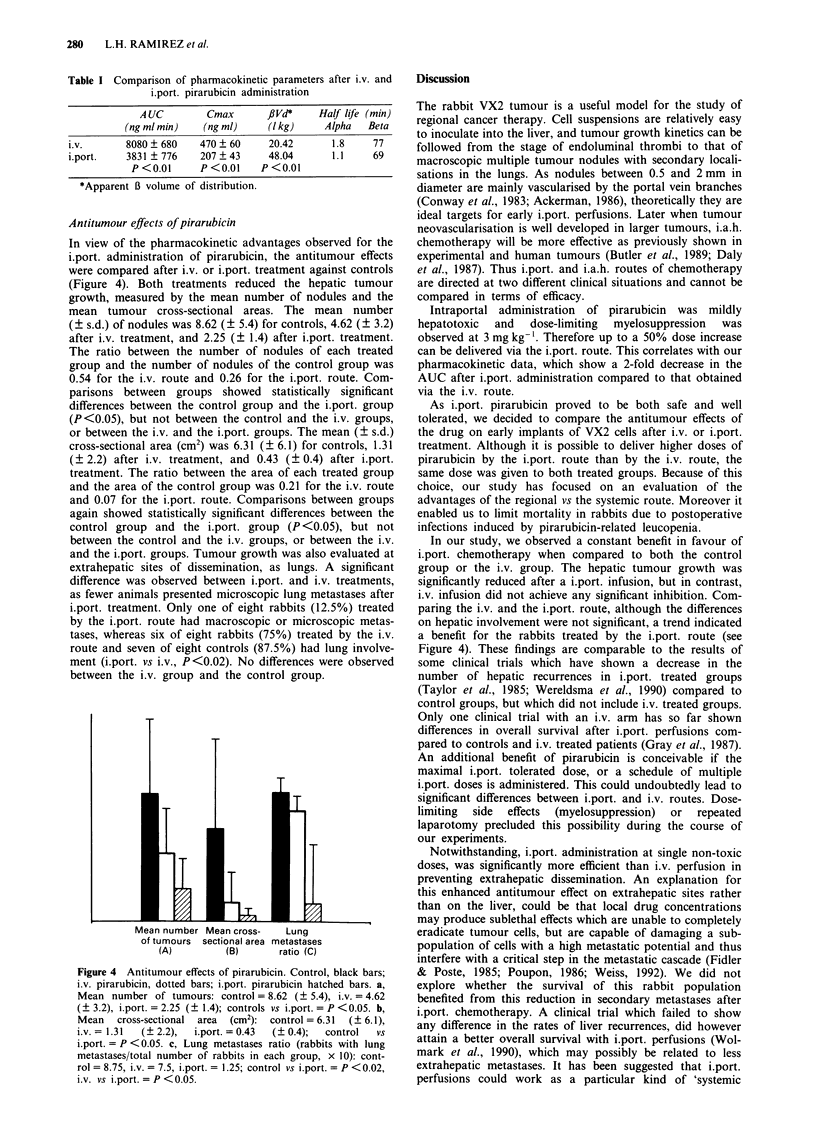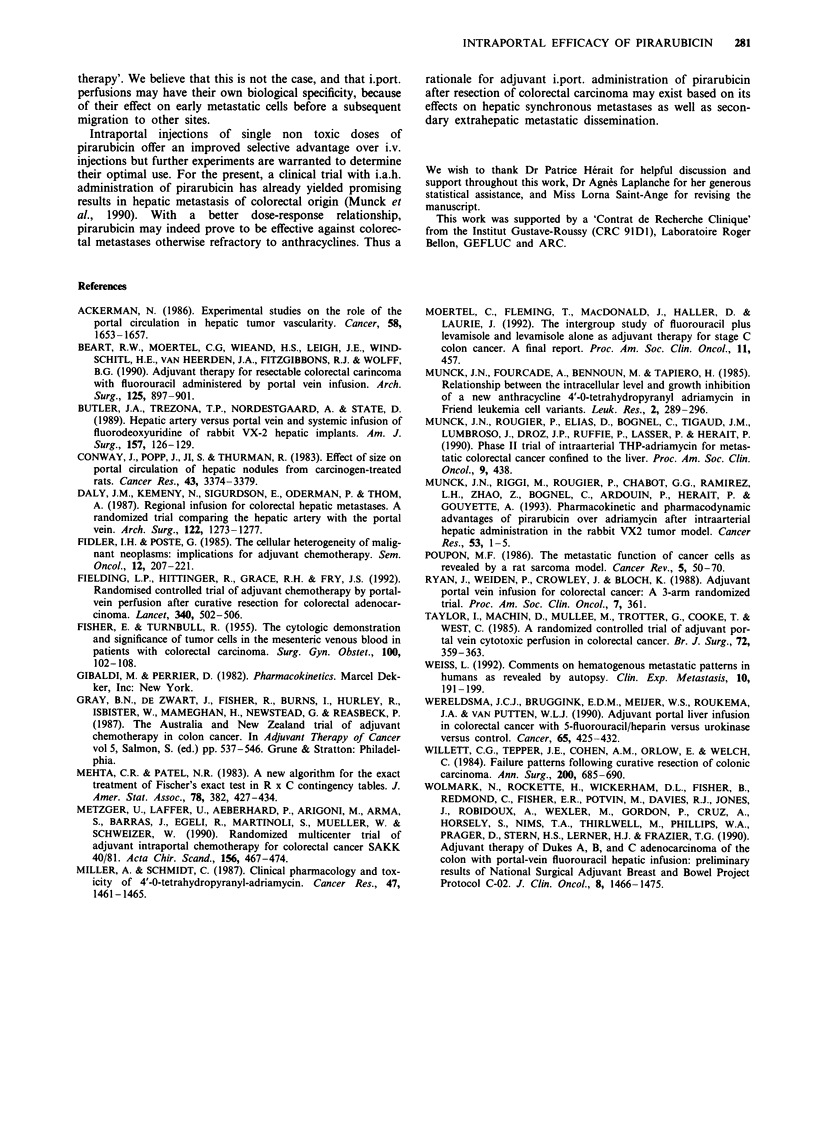# Pharmacology and antitumour effects of intraportal pirarubicin on experimental liver metastases.

**DOI:** 10.1038/bjc.1993.328

**Published:** 1993-08

**Authors:** L. H. Ramirez, J. N. Munck, C. Bognel, Z. Zhao, P. Ardouin, M. F. Poupon, A. Gouyette, P. Rougier

**Affiliations:** Département de Médecine, Institut Gustave-Roussy, Villejuif, France.

## Abstract

**Images:**


*Br. J. Cancer* (1993), **68**, 277–281
© Macmillan Press Ltd., 1993

### Pharmacology and antitumour effects of intraportal pirarubicin on experimental liver metastases

L.H. Ramirez[1], J.-N. Munck[1], C. Bognel[2], Z. Zhao[1], P. Ardouin[3], M.-F. Poupon[4], A. Gouyette[5] & P. Rougier[1]

[1]*Département de Médecine;* [2]*Département d'Anatomopathologie;* [3]*Service d'Expérimentation Animale;* [5]*Laboratoire de Pharmacologie Clinique U 140 INSERM and URA 147 CNRS, Institut Gustave-Roussy, 94805 Villejuif, France and* [4]*URA 620 CNRS Institut Curie, 75005 Paris, France.*

**Summary** Early liver metastases have a predominant portal blood supply. Intraportal (i.port.) vein administration of cytotoxics could theoretically achieve enhanced drug concentrations in tumour cells and be effective as adjuvant therapy after resection of colorectal carcinoma. Pirarubicin (which has a higher hepatic extraction than doxorubicin) was investigated on liver metastases of the VX2 rabbit tumour, which were of less than 2 mm in diameter 7 days after cells injection into the portal vein. To evaluate antitumour activity, 24 rabbits were randomised into three groups 7 days after implantation: (a) control, (b) i.v. pirarubicin, (c) i.port. pirarubicin at doses of 2 mg $kg^{-1}$ in both groups. Portal infusions led to no hematological or hepatic toxicity. Pharmacokinetic parameters showed a significantly reduced systemic exposure after i.port. administration. Fourteen days after treatment, livers and lungs were analysed. The mean number (± s.d.) of tumour foci was (a) 8.62 (± 5.4), (b) 4.62 (± 3.2), (c) 2.25 (± 1.4) ($P<0.05$ a *vs* c). The mean tumour area was (a) 6.31 (± 6.1), (b) 1.31 (± 2.2), (c) 0.43 (± 0.4 $cm^2$) ($P<0.05$ a *vs* c) and the percentage (95% C.I.) of rabbits with lung metastasis was: (a) 87.5% (47–99%), (b) 75% (35–97%), (c) 12.5% (3–52%) ($P<0.02$ b *vs* c). Intraportal pirarubicin seems to be well tolerated and more efficient than i.v. administration, particularly in preventing extrahepatic dissemination.

Residual cancer cells are responsible for treatment failures after curative resection for colorectal cancer and the liver is the most frequent site of relapse (Willett *et al.*, 1984). Systemic post-operative adjuvant therapy based on fluoropyrimidines can reduce the recurrence rates and increase overall survival, as recently observed in Dukes C stage colon cancer (Moertel *et al.*, 1992). With new active drugs and a more efficient targeting of tumour tissues, further improvements could be obtained.

Colon cancer cells disseminate through the mesenteric veins and portal system (Fisher & Turnbull, 1955), and the newly growing liver metastases receive their blood supply from the portal branches, until they develop a main arterial vascularisation (Conway *et al.*, 1983; Ackerman, 1986). Factors such as intraoperative manipulation of the tumour, perioperative impaired immunity and stress, have been reported to facilitate the dissemination and seeding of malignant cells. Regional post-operative adjuvant therapy via the portal vein could be an effective means of preventing the development of liver metastases, by delivering higher local drug concentrations to tumour cells at the onset of metastatic invasion. Several clinical trials have been designed to test this hypothesis (Taylor *et al.*, 1985; Gray *et al.*, 1987; Ryan *et al.*, 1988; Wolmark *et al.*, 1990; Metzger *et al.*, 1990; Wereldsma *et al.*, 1990; Beart *et al.*, 1990; Fielding *et al.*, 1992). Most of them used 5-FU alone, or in association with mitomycin C in different schedules. Current results are controversial and remain inconclusive. Some trials have shown a reduction in the number of hepatic recurrences with no benefit for overall survival (Wereldsma *et al.*, 1990). Others have demonstrated an increase in overall and disease free survival, although no differences were noted in the number of hepatic recurrences (Wolmark *et al.*, 1990).

Other antitumoural compounds could be tested for regional therapy, focusing on drugs with a high hepatic extraction and limited heptatic toxicity. Anthracyclines, are generally considered inactive against colorectal cancer, but pirarubicin, a new derivative, has been recently investigated in pre-clinical and clinical studies (Miller & Schmidt, 1987). This compound has a faster cellular uptake than that of doxorubicin (Munck *et al.*, 1985), as it is much more lipophilic. Indeed, the apparent partition coefficient ($P_{app}$) between octanol and phosphate buffer at pH 7 is 35.8 (log P: 1.55) for pirarubicin, and 0.26 (log P: − 0.59) for doxorubicin (unpublished data). Furthermore, pirarubicin has demonstrated its superiority over doxorubicin in an experimental study with intraarterial hepatic (i.a.h.) administration which appeared essentially due to a greater tumour drug uptake (Munck *et al.*, 1993). Subsequent clinical studies have further demonstrated a high degree of activity, even against colorectal hepatic metastases (Munck *et al.*, 1990). This prompted our investigation of the putative benefit of intraportal infusion of adjuvant pirarubicin in the experimental VX2 tumour in the rabbit.

The present study, on a model of early experimental liver metastases, compares intraportal (i.port.) *vs* intravenous (i.v.) adjuvant infusion of pirarubicin, taking into account both pharmacokinetic parameters and the effects of this cytotoxic on hepatic and extrahepatic tumoural growth and dissemination.

### Materials and methods

### *Animals and anaesthesia*

Female New Zealand white rabbits weighing 2.7–3.2 kg were used (Elevage Scientifique des Dombes, Romans, France). The rabbits were maintained under standard conditions on a laboratory diet and water *ad libitum*. All procedures were carried out under general i.v. anaesthesia using ketamine hydrochloride (50 mg $kg^{-1}$; Ketamine®, Parke Davis) and xilazine 2% (0.1 ml $kg^{-1}$; Rompun®, Bayer). All experiments were conducted in accordance with the European Council directive 86/609/CEE, and French legislation concerning animal welfare.

### *Drugs and chemicals*

Doxorubicin hydrochloride, daunorubicin, doxorubicinol, doxorubicinone, pirarubicinol, and pirarubicin hydrochloride

Correspondence: J.-N. Munck, Institut Gustave-Roussy, rue Camille-Desmoulins, 94805 Villejuif Cedex, France.
Received 26 October 1992; and in revised form 31 March 1993.

were provided by Laboratoire Roger Bellon (Neuilly-sur-Seine, France). The chemical structure of doxorubicin and pirarubicin is depicted in Figure 1. Solvents used for extraction and high performance liquid chromatography (HPLC) analyses were all of HPLC grade or of highest available purity.

*VX2 tumour inoculation and surgical procedures*

The VX2 tumor was kindly provided by Dr G. Orth (U190 INSERM, Institut Pasteur, Paris, France) and was maintained by serial passages in carrier rabbits. A VX2 tumour was removed from one animal, minced in NCTC 109 medium (Eurobio, Paris, France) and filtered through a cotton gauze. The filtrate was adjusted to $3 \times 10^7$ cells $ml^{-1}$ with the above medium containing 10% dimethylsulfoxide (DMSO) and 20% foetal calf serum (Gibco, Paris, France) to constitute a homogeneous stock that was frozen in liquid nitrogen in 1 ml aliquots and used throughout the study. This procedure minimised inter-subject variations in tumour growth rate.

Hepatic implantation of the VX2 carcinoma was accomplished through a small median subxyphoid incision. A 1 ml frozen sample of VX2 cells was rapidly thawed and centrifuged. The cell pellet was adjusted to 0.5 ml (NCTC 109 medium), and injected with a 24-gauge catheter into the main portal vein.

*Establishment of a model of early hepatic metastases*

In order to establish the optimal time for i.port. drug administration, the kinetics of VX2 tumour growth after i.port. inoculation were determined. For this purpose three groups of four rabbits each were studied, and the animals were sacrificed at 7, 14, and 21 days after VX2 cell injections. Livers were cut into 5 mm slices, and the number of tumours and their diameters were recorded and cross-sectional areas calculated. Lung slides were screened for the presence or absence of metastases.

*Drug administration and collection of biological samples*

Intraportal perfusions of pirarubicin were done with a 24-gauge catheter which was inserted into the portal vein. The drug was infused over 5 min with a pump (MS 16 A Graseby, Michel Frères, Montreuil, France). Intravenous perfusions were done through the auricular vein with the same pump, and a sham laparotomy was performed so that operative stress was similar to that of the portal perfused group.

Doses of 2 mg $kg^{-1}$ of pirarubicin were administered for pharmacokinetic studies. Heparinised blood samples (2 ml) were drawn from the left ear artery prior to injection, and at 0.5, 2, 5, 15, 30 and 60 min thereafter. Samples were centrifuged (2000 *g*, 10 min) and the plasma samples were frozen at − 20°C until HPLC analysis.

To assess local liver toxicity, alanine aminotransferase (ALT), aspartate aminotransferase (AST), alkaline phosphatase, and total bilirubin levels were determined before pirarubicin perfusions and 2 and 7 days thereafter. Hematological toxicity was evaluated according to blood cell counts at 5, 7 and 14 days, which were compared to pretreatment baseline values.

*Determination of maximal tolerated single i.port. doses of pirarubicin*

The maximal tolerated dose (MTD) in rabbits for the i.v. route had been previously determined at 2 mg $kg^{-1}$ (unpublished data). Higher i.v. doses (2.5 mg $kg^{-1}$) were associated with lethal toxicity. The determination of the MTD for the i.port. route was achieved by escalating the 2 mg $kg^{-1}$ dose by 0.5 mg increments. Pirarubicin was administered at single doses of 2 mg, 2.5 mg, 3 mg, and 3.5 mg to four groups of four rabbits without a tumour graft.

*Determination of pirarubicin plasma concentration*

Plasma concentrations were determined using reversed-phase HPLC. Daunorubicin was added as the internal standard (100 ng $ml^{-1}$). Half a ml of plasma was extracted on 100 mg octadecyl (C18) columns (1 ml Bakerbond spe®, Baker, Phillipsburg, NJ) preconditioned with 1 ml of methanol, followed by 1 ml of water. After air drying, elution was accomplished with 1 ml of methanol/dichloromethane (1:1, v:v) following the addition of 200 µl of DMSO. The volume was then reduced to approximately 200 µl under a nitrogen stream before HPLC injection. This procedure allowed a 95% recovery of pirarubicin, internal standard and metabolites. The HPLC system consisted of a C18 column (Nucleosil C, 10 µm, 3.9 × 300 mm, SFCC, Neuilly-sur-Seine, France), a Wisp automatic injector (710B, Waters Associated, Milford, Ma, USA), a 6000A pump (Waters), and a fluorescence detector (Shoeffel FS 970) set at 251 nm (ex.) and 550 nm (em.). The mobile phase consisted of water (adjusted to pH 2.4 with phosphoric acid) and acetonitrile (68:32, v:v) at a flow rate of 1.75 ml $min^{-1}$. Under these conditions, the retention times of doxorubicinol, doxorubicin, pirarubicinol, daunorubicin, and pirarubicin were 3.59, 4.48, 5.94, 6.90 and 9.65 min, respectively. Two peaks corresponding to doxorubicinol and doxorubicin were observed in plasma after doxorubicin injection, whereas four peaks were detected following pirarubicin infusion. These peaks coeluted with doxorubicinol, doxorubicin, pirarubicinol and pirarubicin.

*Pharmacokinetic analysis*

Plasma concentrations were best fitted to a two-compartment model with first-order elimination using a 5 min i.v. infusion input. Curve fitting was accomplished with the PC-NONLIN nonlinear regression program (Statistical Consultants Inc., Lexington, KY) using a data weight of the reciprocal of the concentration. The total area under the curve (AUC) was determined using the trapezoidal method. The other pharmacokinetic parameters were calculated according to standard methods (Gibaldi & Perrier, 1982).

*Antitumour effects of intravenous and intraportal pirarubicin*

Eight consecutive experiments were conducted on groups of three rabbits each in a block-design experiment. They were inoculated and randomised to receive: no treatment, controls

Daunorubicin:: R=H
Doxorubicin:: R=OH

Pirarubicin

**Figure 1** Chemical structure of doxorubicin and pirarubicin.

(group A), i.v. pirarubicin $2 mg kg^{-1}$ (group B), and i.port. pirarubicin $2 mg kg^{-1}$ (group C). Both treatments were administered in single doses 7 days after tumour inoculation. Fourteen days later, the rabbits were sacrificed for histological study. Hepatic tumour invasion was analysed by the number of nodules present at 21 days and by their cross-sectional area. Extrahepatic dissemination was evaluated by the presence or absence of lung metastases in each group. A lung metastasis ratio was defined as the ratio between the number of rabbits with lung metastases and the total number of rabbits in each group.

### *Statistical analysis*

Biological and pharmacokinetic results were compared using the Student's *t*-test. The number of liver nodules and tumour areas were compared after transformation of each measure so that $Y = \sqrt{x}$ for hepatic nodes, and $Y = \log x$ for cross-sectional areas in order to approximate to normal probability distributions and equalise variances.

Analysis of data for lung metastases was done with Pearson's exact chi squared test (Mehta & Patel, 1983), and Fisher test 2 × 2. Significance was assumed for all tests at $P<0.05$.

### Results

### *Experimental model*

Operative mortality in rabbits due to surgery or anaesthesia was approximately 10%, and successful grafting was obtained in 92.3% of cases. Tumour nodules were not yet macroscopically detectable at 7 days, but microscopic analysis of livers showed the presence of tumourous thrombi starting to extend from the portal vein (Figure 2). No lung metastases were observed. On day 14, two of five rabbits presented macroscopic nodules with diameters exceeding 5 mm, and one had a lung metastasis. On day 21 all rabbits had macroscopic tumours and three of four rabbits already had lung metastases. These data justified (in accordance with our study objectives) the choice of day 7 for studying the effects of an early adjuvant treatment with pirarubicin.

### *Tolerance of i.port. pirarubicin*

After i.v. administration, the maximal tolerated dose is $2 mg kg^{-1}$. Doses above $2 mg kg^{-1}$ caused severe hematological toxicity, and $3 mg kg^{-1}$ was invariably lethal. After i.port. administration, hepatic toxicity only occurred at doses of $3.5 mg kg^{-1}$ with the onset of mild centrolobular hepatic necrosis and periportal fibrosis with limited and reversible increases (2 ×) of ALT and AST. Lower doses did neither produce alterations of liver tests, nor induce histologic modifications. However i.port. administration had to be limited at $3 mg kg^{-1}$ because of leucopenia. In order to compare the same dose of i.v. and i.port. pirarubicin, and to avoid the risk of mortality with high doses of pirarubicin, we chose a $2 mg kg^{-1}$ dose for all subsequent experiments.

### *Pharmacokinetics*

Plasma concentrations of pirarubicin following either i.v. or i.port. administration are depicted in Figure 3. In all cases, plasma decay was biphasic and fitted to a two-compartment model. Intraportal pirarubicin infusion led to a significant 2-fold decrease in both the peak plasma concentration of pirarubicin (Cmax), and the AUC, when compared to the i.v. route (Table I). Plasma metabolite levels (doxorubicin, doxorubicinol, pirarubicinol) remained constantly low, and no significant difference was observed between the two routes of administration (data not shown).

**Figure 3** Plasma concentrations of pirarubicin in rabbits following an intravenous (i.v.) or intraportal (i.port.) administration of a 2 mg kg dose. Concentrations were determined by HPLC. ○, i.v.; ●, i.port. Each point represents the mean of measurements (± s.e.) done in eight rabbits.

**Figure 2** Liver specimen of a rabbit sacrificed 7 days after intraportal injection of VX2 cancer cells. The arrow points to a neoplastic intraportal thrombus.

**Table I** Comparison of pharmacokinetic parameters after i.v. and i.port. pirarubicin administration

| | *AUC (ng ml min)* | *Cmax (ng ml)* | *ßVd* (l kg)* | *Half life (min) Alpha* | *Beta* |
|---|---|---|---|---|---|
| i.v. | 8080 ± 680 | 470 ± 60 | 20.42 | 1.8 | 77 |
| i.port. | 3831 ± 776 | 207 ± 43 | 48.04 | 1.1 | 69 |
| | $P<0.01$ | $P<0.01$ | $P<0.01$ | | |

*Apparent ß volume of distribution.

*Antitumour effects of pirarubicin*

In view of the pharmacokinetic advantages observed for the i.port. administration of pirarubicin, the antitumour effects were compared after i.v. or i.port. treatment against controls (Figure 4). Both treatments reduced the hepatic tumour growth, measured by the mean number of nodules and the mean tumour cross-sectional areas. The mean number (± s.d.) of nodules was 8.62 (± 5.4) for controls, 4.62 (± 3.2) after i.v. treatment, and 2.25 (± 1.4) after i.port. treatment. The ratio between the number of nodules of each treated group and the number of nodules of the control group was 0.54 for the i.v. route and 0.26 for the i.port. route. Comparisons between groups showed statistically significant differences between the control group and the i.port. group ($P<0.05$), but not between the control and the i.v. groups, or between the i.v. and the i.port. groups. The mean (± s.d.) cross-sectional area ($cm^2$) was 6.31 (± 6.1) for controls, 1.31 (± 2.2) after i.v. treatment, and 0.43 (± 0.4) after i.port. treatment. The ratio between the area of each treated group and the area of the control group was 0.21 for the i.v. route and 0.07 for the i.port. route. Comparisons between groups again showed statistically significant differences between the control group and the i.port. group ($P<0.05$), but not between the control and the i.v. groups, or between the i.v. and the i.port. groups. Tumour growth was also evaluated at extrahepatic sites of dissemination, as lungs. A significant difference was observed between i.port. and i.v. treatments, as fewer animals presented microscopic lung metastases after i.port. treatment. Only one of eight rabbits (12.5%) treated by the i.port. route had macroscopic or microscopic metastases, whereas six of eight rabbits (75%) treated by the i.v. route and seven of eight controls (87.5%) had lung involvement (i.port. *vs* i.v., $P<0.02$). No differences were observed between the i.v. group and the control group.

**Figure 4** Antitumour effects of pirarubicin. Control, black bars; i.v. pirarubicin, dotted bars; i.port. pirarubicin hatched bars. **a**, Mean number of tumours: control = 8.62 (± 5.4), i.v. = 4.62 (± 3.2), i.port. = 2.25 (± 1.4); controls *vs* i.port. = $P<0.05$. **b**, Mean cross-sectional area ($cm^2$): control = 6.31 (± 6.1), i.v. = 1.31 (± 2.2), i.port. = 0.43 (± 0.4); control *vs* i.port. = $P<0.05$. **c**, Lung metastases ratio (rabbits with lung metastases/total number of rabbits in each group, × 10): control = 8.75, i.v. = 7.5, i.port. = 1.25; control *vs* i.port. = $P<0.02$, i.v. *vs* i.port. = $P<0.05$.

### Discussion

The rabbit VX2 tumour is a useful model for the study of regional cancer therapy. Cell suspensions are relatively easy to inoculate into the liver, and tumour growth kinetics can be followed from the stage of endoluminal thrombi to that of macroscopic multiple tumour nodules with secondary localisations in the lungs. As nodules between 0.5 and 2 mm in diameter are mainly vascularised by the portal vein branches (Conway *et al.*, 1983; Ackerman, 1986), theoretically they are ideal targets for early i.port. perfusions. Later when tumour neovascularisation is well developed in larger tumours, i.a.h. chemotherapy will be more effective as previously shown in experimental and human tumours (Butler *et al.*, 1989; Daly *et al.*, 1987). Thus i.port. and i.a.h. routes of chemotherapy are directed at two different clinical situations and cannot be compared in terms of efficacy.

Intraportal administration of pirarubicin was mildly hepatotoxic and dose-limiting myelosuppression was observed at 3 mg $kg^{-1}$. Therefore up to a 50% dose increase can be delivered via the i.port. route. This correlates with our pharmacokinetic data, which show a 2-fold decrease in the AUC after i.port. administration compared to that obtained via the i.v. route.

As i.port. pirarubicin proved to be both safe and well tolerated, we decided to compare the antitumour effects of the drug on early implants of VX2 cells after i.v. or i.port. treatment. Although it is possible to deliver higher doses of pirarubicin by the i.port. route than by the i.v. route, the same dose was given to both treated groups. Because of this choice, our study has focused on an evaluation of the advantages of the regional *vs* the systemic route. Moreover it enabled us to limit mortality in rabbits due to postoperative infections induced by pirarubicin-related leucopenia.

In our study, we observed a constant benefit in favour of i.port. chemotherapy when compared to both the control group or the i.v. group. The hepatic tumour growth was significantly reduced after a i.port. infusion, but in contrast, i.v. infusion did not achieve any significant inhibition. Comparing the i.v. and the i.port. route, although the differences on hepatic involvement were not significant, a trend indicated a benefit for the rabbits treated by the i.port. route (see Figure 4). These findings are comparable to the results of some clinical trials which have shown a decrease in the number of hepatic recurrences in i.port. treated groups (Taylor *et al.*, 1985; Wereldsma *et al.*, 1990) compared to control groups, but which did not include i.v. treated groups. Only one clinical trial with an i.v. arm has so far shown differences in overall survival after i.port. perfusions compared to controls and i.v. treated patients (Gray *et al.*, 1987). An additional benefit of pirarubicin is conceivable if the maximal i.port. tolerated dose, or a schedule of multiple i.port. doses is administered. This could undoubtedly lead to significant differences between i.port. and i.v. routes. Dose-limiting side effects (myelosuppression) or repeated laparotomy precluded this possibility during the course of our experiments.

Notwithstanding, i.port. administration at single non-toxic doses, was significantly more efficient than i.v. perfusion in preventing extrahepatic dissemination. An explanation for this enhanced antitumour effect on extrahepatic sites rather than on the liver, could be that local drug concentrations may produce sublethal effects which are unable to completely eradicate tumour cells, but are capable of damaging a subpopulation of cells with a high metastatic potential and thus interfere with a critical step in the metastatic cascade (Fidler & Poste, 1985; Poupon, 1986; Weiss, 1992). We did not explore whether the survival of this rabbit population benefited from this reduction in secondary metastases after i.port. chemotherapy. A clinical trial which failed to show any difference in the rates of liver recurrences, did however attain a better overall survival with i.port. perfusions (Wolmark *et al.*, 1990), which may possibly be related to less extrahepatic metastases. It has been suggested that i.port. perfusions could work as a particular kind of 'systemic therapy'. We believe that this is not the case, and that i.port. perfusions may have their own biological specificity, because of their effect on early metastatic cells before a subsequent migration to other sites.

Intraportal injections of single non toxic doses of pirarubicin offer an improved selective advantage over i.v. injections but further experiments are warranted to determine their optimal use. For the present, a clinical trial with i.a.h. administration of pirarubicin has already yielded promising results in hepatic metastasis of colorectal origin (Munck *et al.*, 1990). With a better dose-response relationship, pirarubicin may indeed prove to be effective against colorectal metastases otherwise refractory to anthracyclines. Thus a rationale for adjuvant i.port. administration of pirarubicin after resection of colorectal carcinoma may exist based on its effects on hepatic synchronous metastases as well as secondary extrahepatic metastatic dissemination.

We wish to thank Dr Patrice Hérait for helpful discussion and support throughout this work, Dr Agnès Laplanche for her generous statistical assistance, and Miss Lorna Saint-Ange for revising the manuscript.

This work was supported by a 'Contrat de Recherche Clinique' from the Institut Gustave-Roussy (CRC 91D1), Laboratoire Roger Bellon, GEFLUC and ARC.

### References

ACKERMAN, N. (1986). Experimental studies on the role of the portal circulation in hepatic tumor vascularity. *Cancer*, **58**, 1653-1657.

BEART, R.W., MOERTEL, C.G, WIEAND, H.S., LEIGH, J.E., WINDSCHITL, H.E., VAN HEERDEN, J.A., FITZGIBBONS, R.J. & WOLFF, B.G. (1990). Adjuvant therapy for resectable colorectal carincoma with fluorouracil administered by portal vein infusion. *Arch. Surg.*, **125**, 897-901.

BUTLER, J.A., TREZONA, T.P., NORDESTGAARD, A. & STATE, D. (1989). Hepatic artery versus portal vein and systemic infusion of fluorodeoxyuridine of rabbit VX-2 hepatic implants. *Am. J. Surg.*, **157**, 126-129.

CONWAY, J., POPP, J., JI, S. & THURMAN, R. (1983). Effect of size on portal circulation of hepatic nodules from carcinogen-treated rats. *Cancer Res.*, **43**, 3374-3379.

DALY, J.M., KEMENY, N., SIGURDSON, E., ODERMAN, P. & THOM, A. (1987). Regional infusion for colorectal hepatic metastases. A randomized trial comparing the hepatic artery with the portal vein. *Arch. Surg.*, **122**, 1273-1277.

FIDLER, I.H. & POSTE, G. (1985). The cellular heterogeneity of malignant neoplasms: implications for adjuvant chemotherapy. *Sem. Oncol.*, **12**, 207-221.

FIELDING, L.P., HITTINGER, R., GRACE, R.H. & FRY, J.S. (1992). Randomised controlled trial of adjuvant chemotherapy by portal-vein perfusion after curative resection for colorectal adenocarcinoma. *Lancet*, **340**, 502-506.

FISHER, E. & TURNBULL, R. (1955). The cytologic demonstration and significance of tumor cells in the mesenteric venous blood in patients with colorectal carcinoma. *Surg. Gyn. Obstet.*, **100**, 102-108.

GIBALDI, M. & PERRIER, D. (1982). *Pharmacokinetics*. Marcel Dekker, Inc: New York.

GRAY, B.N., DE ZWART, J., FISHER, R., BURNS, I., HURLEY, R., ISBISTER, W., MAMEGHAN, H., NEWSTEAD, G. & REASBECK, P. (1987). The Australia and New Zealand trial of adjuvant chemotherapy in colon cancer. In *Adjuvant Therapy of Cancer* vol 5, Salmon, S. (ed.) pp. 537-546. Grune & Stratton: Philadelphia.

MEHTA, C.R. & PATEL, N.R. (1983). A new algorithm for the exact treatment of Fischer's exact test in R x C contingency tables. *J. Amer. Stat. Assoc.*, **78**, 382, 427-434.

METZGER, U., LAFFER, U., AEBERHARD, P., ARIGONI, M., ARMA, S., BARRAS, J., EGELI, R., MARTINOLI, S., MUELLER, W. & SCHWEIZER, W. (1990). Randomized multicenter trial of adjuvant intraportal chemotherapy for colorectal cancer SAKK 40/81. *Acta Chir. Scand.*, **156**, 467-474.

MILLER, A. & SCHMIDT, C. (1987). Clinical pharmacology and toxicity of 4'-0-tetrahydropyranyl-adriamycin. *Cancer Res.*, **47**, 1461-1465.

MOERTEL, C., FLEMING, T., MACDONALD, J., HALLER, D. & LAURIE, J. (1992). The intergroup study of fluorouracil plus levamisole and levamisole alone as adjuvant therapy for stage C colon cancer. A final report. *Proc. Am. Soc. Clin. Oncol.*, **11**, 457.

MUNCK, J.N., FOURCADE, A., BENNOUN, M. & TAPIERO, H. (1985). Relationship between the intracellular level and growth inhibition of a new anthracycline 4'-0-tetrahydropyranyl adriamycin in Friend leukemia cell variants. *Leuk. Res.*, **2**, 289-296.

MUNCK, J.N., ROUGIER, P., ELIAS, D., BOGNEL, C., TIGAUD, J.M., LUMBROSO, J., DROZ, J.P., RUFFIE, P., LASSER, P. & HERAIT, P. (1990). Phase II trial of intraarterial THP-adriamycin for metastatic colorectal cancer confined to the liver. *Proc. Am. Soc. Clin. Oncol.*, **9**, 438.

MUNCK, J.N., RIGGI, M., ROUGIER, P., CHABOT, G.G., RAMIREZ, L.H., ZHAO, Z., BOGNEL, C., ARDOUIN, P., HERAIT, P. & GOUYETTE, A. (1993). Pharmacokinetic and pharmacodynamic advantages of pirarubicin over adriamycin after intraarterial hepatic administration in the rabbit VX2 tumor model. *Cancer Res.*, **53**, 1-5.

POUPON, M.F. (1986). The metastatic function of cancer cells as revealed by a rat sarcoma model. *Cancer Rev.*, **5**, 50-70.

RYAN, J., WEIDEN, P., CROWLEY, J. & BLOCH, K. (1988). Adjuvant portal vein infusion for colorectal cancer: A 3-arm randomized trial. *Proc. Am. Soc. Clin. Oncol.*, **7**, 361.

TAYLOR, I., MACHIN, D., MULLEE, M., TROTTER, G., COOKE, T. & WEST, C. (1985). A randomized controlled trial of adjuvant portal vein cytotoxic perfusion in colorectal cancer. *Br. J. Surg.*, **72**, 359-363.

WEISS, L. (1992). Comments on hematogenous metastatic patterns in humans as revealed by autopsy. *Clin. Exp. Metastasis*, **10**, 191-199.

WERELDSMA, J.C.J., BRUGGINK, E.D.M., MEIJER, W.S., ROUKEMA, J.A. & VAN PUTTEN, W.L.J. (1990). Adjuvant portal liver infusion in colorectal cancer with 5-fluorouracil/heparin versus urokinase versus control. *Cancer*, **65**, 425-432.

WILLETT, C.G., TEPPER, J.E., COHEN, A.M., ORLOW, E. & WELCH, C. (1984). Failure patterns following curative resection of colonic carcinoma. *Ann. Surg.*, **200**, 685-690.

WOLMARK, N., ROCKETTE, H., WICKERHAM, D.L., FISHER, B., REDMOND, C., FISHER, E.R., POTVIN, M., DAVIES, R.J., JONES, J., ROBIDOUX, A., WEXLER, M., GORDON, P., CRUZ, A., HORSELY, S., NIMS, T.A., THIRLWELL, M., PHILLIPS, W.A., PRAGER, D., STERN, H.S., LERNER, H.J. & FRAZIER, T.G. (1990). Adjuvant therapy of Dukes A, B, and C adenocarcinoma of the colon with portal-vein fluorouracil hepatic infusion: preliminary results of National Surgical Adjuvant Breast and Bowel Project Protocol C-02. *J. Clin. Oncol.*, **8**, 1466-1475.